# Transcriptomic analysis of genetically defined autism candidate genes reveals common mechanisms of action

**DOI:** 10.1186/2040-2392-4-45

**Published:** 2013-11-15

**Authors:** Thomas A Lanz, Edward Guilmette, Mark M Gosink, James E Fischer, Lawrence W Fitzgerald, Diane T Stephenson, Mathew T Pletcher

**Affiliations:** 1 Neuroscience Research Unit, Pfizer Inc, Main Street, Cambridge, MA 02140, USA; 2 Investigative Toxicology, Pfizer, Inc, Eastern Point Road, Groton, CT 06340, USA; 3 Compound Safety Prediction, Pfizer, Inc, Eastern Point Road, Groton, CT 06340, USA; 4 Rare Disease Research Unit, Pfizer, Inc, Cambridge Park Drive, Cambridge, MA 02140, USA; 5 Current Address: LW Fitzgerald Consulting, LLC, 35 Plum Hill Rd., East Lyme, CT 06333, USA; 6 Current Address: Critical Path Institute, East River Road, Tucson, AZ 85718, USA

## Abstract

**Background:**

Austism spectrum disorder (ASD) is a heterogeneous behavioral disorder or condition characterized by severe impairment of social engagement and the presence of repetitive activities. The molecular etiology of ASD is still largely unknown despite a strong genetic component. Part of the difficulty in turning genetics into disease mechanisms and potentially new therapeutics is the sheer number and diversity of the genes that have been associated with ASD and ASD symptoms. The goal of this work is to use shRNA-generated models of genetic defects proposed as causative for ASD to identify the common pathways that might explain how they produce a core clinical disability.

**Methods:**

Transcript levels of *Mecp2*, *Mef2a*, *Mef2d*, *Fmr1*, *Nlgn1*, *Nlgn3*, *Pten*, and *Shank3* were knocked-down in mouse primary neuron cultures using shRNA constructs. Whole genome expression analysis was conducted for each of the knockdown cultures as well as a mock-transduced culture and a culture exposed to a lentivirus expressing an anti-luciferase shRNA. Gene set enrichment and a causal reasoning engine was employed to identify pathway level perturbations generated by the transcript knockdown.

**Results:**

Quantification of the shRNA targets confirmed the successful knockdown at the transcript and protein levels of at least 75% for each of the genes. After subtracting out potential artifacts caused by viral infection, gene set enrichment and causal reasoning engine analysis showed that a significant number of gene expression changes mapped to pathways associated with neurogenesis, long-term potentiation, and synaptic activity.

**Conclusions:**

This work demonstrates that despite the complex genetic nature of ASD, there are common molecular mechanisms that connect many of the best established autism candidate genes. By identifying the key regulatory checkpoints in the interlinking transcriptional networks underlying autism, we are better able to discover the ideal points of intervention that provide the broadest efficacy across the diverse population of autism patients.

## Background

Autism spectrum disorder (ASD) is a heterogeneous developmental disease that is primarily characterized by behavioral and social impairments such as the presence of repetitive or ritualistic activities, social withdrawal, and difficulty with proper communication. ASD is more commonly diagnosed in male individuals at a 4:1 ratio and its incidence has notably risen over time. It is currently estimated that ASD afflicts up to one out of every eighty-eight individuals and is now counted as the second most common developmental disability after intellectual disability [1-3]. Current treatment options for autism are limited, focusing primarily on behavioral therapies and repurposed drugs whose primary indication is not autism.

It is long been appreciated that ASD has a strong genetic component underlying its etiology. Early twin studies, examining the co-inheritance of ASD among monozygotic twins, reported a heritability rate for ASD between 60% and 90% [4]. The role of genetics in ASD has been further elucidated and refined at the single gene level as tools such as genome-wide association studies (GWAS), copy number variant (CNV) mapping, and whole exome/genome sequencing have been applied to the disease [5-10]. A clear association has been demonstrated between genetic variants in genes, such as *Contactin-associated protein-like 2* (*Cntnap2*) and *Semaphorin-5A* (*Sema5A*), and ASD, and the localization of rare deletions and duplications has not only led to the identification of new autism candidate genes, such as *SH3 and multiple ankyrin repeat domains 3* (*Shank3*), but also the creation of new mouse models that parallel ASD at both the genetic and behavioral level [11-14].

Our understanding of the genetics and molecular mechanisms of ASD has also been greatly enriched by the study of rare diseases caused by mutations in a well-defined single gene with symptomatic overlap with ASD. Two of the best known examples of this are Fragile X and Rett syndromes. Fragile X is caused by an expansion of a CGG repeat in the Fragile X mental retardation-1 (*Fmr1*) gene and results in mental retardation. Fragile X, because it is X-linked, is preferentially found in male individuals and 25% to 33% of Fragile X patients also meet the criteria for ASD, making it one of the most common genetic causes of autism [15]. Rett Syndrome is also X-linked but unlike Fragile X and ASD, it is predominantly diagnosed in female individuals, because the hemizygous state is often lethal. Rett syndrome too is marked by mental retardation and frequent comorbidity with autism. In addition to being directly tied to ASD through Rett, *Methyl-CpG binding protein 2 (Mecp2)*, a transcription factor mutated in Rett, regulates the expression of other genes that have been tied to ASD, including *Brain-derived neurotrophic factor* (*Bdnf*) [16,17].

Through the use of modern genetic methods and the study of syndromic forms of autism, over 200 genes have been associated with ASD [18]. In an attempt to gain a better understanding of molecular pathophysiology of the disease, tools such as pathway analysis [19] and protein-protein interaction networks [20-22] have been deployed to identify common mechanisms among these autism-risk genes, and one of the dominant themes that has emerged is a convergence on synapse integrity and dendritic spine formation [23-25]. *Phosphatase and tensin homolog* (*Pten*), the causative gene for Cowden syndrome - another syndromic form of autism - is shown to cause increased neuronal spine density, dysfunction in excitatory and inhibitory synaptic activity and decreased synaptic plasticity when deleted [26-28]. *Shank3* encodes a synaptic scaffolding protein while *Neuroligin 1* and *3* (*Nlgn1, Nlgn3*) produce synaptic cellular adhesion molecules. All three genes have been shown to be altered in ASD patients [29-31]. Finally, *Myocyte enhancer factor 2A* and *2D* (*Mef2A, Mef2D*) are activity-dependent genes that encode transcription factors regulating multiple additional genes implicated in ASD (*Ube3A*, *Slc9A6*, *Pcdh10*, and *C3orf58*) [32], and knockdown of these genes in primary neurons has been shown to regulate synapse density [33].

Despite the clues that have been provided by these genetic links, a true understanding of how those genetic defects translate into altered biology have continued to be elusive and therefore have made the development of new therapies for ASD difficult. The current gross appreciation of impacted dendritic spines and synaptic health falls short of the digital visualization of the molecular mechanisms of ASD necessary to advance the field. Therefore, in this study, we sought to determine the molecular consequences of the loss of function of these diverse genes that have been genetically implicated in autism by use of an *in vitro* model system. Primary neuronal cultures are a well-established model for studying fundamental synaptic biology with a well-characterized trajectory of synaptic differentiation and function [5,34]. These cultures have proven to be a robust system for characterizing the transcriptional consequences of synaptic modulation under a number of settings [32,35,36]. We have focused on cortex as a tissue of origin based on observation of pathologic changes in post-mortem ASD cortex [8] and prior work studying ASD-relevant gene function in cortical neurons [10].

By knocking down *Mecp2*, *Mef2a*, *Mef2d*, *Fmr1*, *Nlgn1*, *Nlgn3*, *Pten*, and *Shank3* (Table 1) in murine primary cortical neurons, we were able to compare and contrast the varying transcriptional profiles of each transcriptional inhibition to arrive at core signaling pathways that unite this otherwise disparate group. Pathways that are in common between the various candidate genes would provide one potential explanation of how a mutation in each them might produce the same clinical outcome - ASD. As all of these genes play a role relevant for synaptic structure or function, the hypothesis was that common downstream genes and pathways might be perturbed. For a disorder with heterogeneous genetic backgrounds that produce common behavioral phenotypes, a common molecular pathway could provide a new avenue for therapeutic intervention.

**Table 1:** Description of genes selected for short-hairpin (sh)RNA knockdown and linkage to ASD

**Gene**	**Function**	**Cellular localization**	**Genetic evidence**	**Reference**
*Methyl CpG binding protein 2 (Mecp2)*	Transcriptional repressor	Nuclear	Causes Rett syndrome, which shares some symptom domains with autism	[37]
*Myocyte-specific enhancer factor 2a (Mef2a)*	Transcription factor	Nuclear	Rare single gene mutation in downstream targets associated with Autism symptom domains	[32]
*Myocyte-specific enhancer factor 2d (Mef2d)*	Transcription factor	Nuclear	Rare single gene mutation in downstream targets associated with Autism symptom domains	[32]
*Fragile X mental retardation 1 (Fmr1)*	RNA binding protein	Nuclear	Causes Fragile X, which shares some symptom domains with autism	[38]
*Neuroligin-1 (Nlgn1)*	Synaptic remodeling	Synaptic	Rare single gene mutation associated with autism symptom domains	[30]
*Neuroligin-3 (Nlgn3)*	Synaptic remodeling	Synaptic	Rare single gene mutation associated with autism symptom domains	[31]
*Phosphatase and tensin homolog (Pten)*	Regulator of the cell cycle	Nuclear and synaptic	Causes Cowden syndrome which shares some symptom domains with autism	[26,39]
*SH3 and multiple ankyrin repeat domains 3 (Shank3)*	Scaffold protein	Synaptic	Rare single gene mutation associated with autism symptom domains	[12,29]

## Methods

### Lentiviral shRNA construct generation and production

Lentiviral constructs were generated by cloning annealed and kinased, complementary oligonucleotides into the lentiviral vector pLL3.7_H1 (a version of pLL3.7, Patrick Stern-MIT, modified to encode human H1 promoter to drive short-hairpin (sh)RNA expression). Individual gene’s target sense sequence (Table 2) followed by the loop sequence TTCAAGAGA, targets’ corresponding antisense and TTTTTT terminator sequences oligos were ligated (New England Biolabs, Ipswich, MA, USA) into the BamHI (5′) and XhoI (3′) cloning sites downstream of the human H1 promoter into pLL3.7_H1.

**Table 2:** shRNA sequences used for knockdown experiments

**Gene**	**GenBank accession number**	**shRNA target sequence**	**TaqMan assay ID**
*Mecp2*	NM_010788	GCCGATCTGCTGGAAAGTATG	Mm00465017_m1
*Mef2a*	NM_001033713	GCCCTAATGCTTTGTCGTACA	Mm00488963_m1
*Mef2d*	NM_133665	GTAGCTCTCTGGTCACTCC	Mm00504929_m1
*Nlgn1*	NM_138666	GCAGATTGCCTGAAGTTATGC	Mm02344307_m1
*Nlgn3*	NM_172932	GGATATGGTGGATTGTCTTCG	Mm01225953_m1
*Pten*	NM_008960	GGACAAGTTCATGTACTTTGA	Mm00477210_m1
*Shank3*	NM_021423	GGGAGAAGTTGGATGAGATCC	Mm00498775_m1
*Gapdh*	NM_008084	Not Applicable	4352339E
*Luciferase* (pGL3-Basic)	U47295	AATTCAGCGGGAGCCACCTGA	Not Applicable

Lentivirus was produced per manufacturer’s instructions via quadruple co-transfection of shRNA-containing pLL3.7_H1 plasmid along with the 3 plasmid ViraPower (Life Technologies, Grand Island, NY, USA) system into HEK293T cells. Then, 24 hours post transfection, the media were changed to complete neurobasal media (Life Technologies) and lentivirus-conditioned media were harvested 48 hours later. Functional titer was determined based on green fluorescent protein (GFP) co-expression in HEK293T cells using flow cytometry (FACscalibur, Becton Dickenson, Franklin Lakes, NJ, USA). Optimal lentiviral transduction of primary cultured cortical neurons was determined to be a multiplicity of infection (MOI) of 3.0, based on fluorescence.

### Primary neuronal cultures and transductions

Mouse primary neuronal cultures were prepared from day-16 C57BL6/J embryos. All procedures related to animal care and treatment were conducted under a protocol approved by the Pfizer Institutional Animal Care and Use Committee, according to the guidelines of the National Research Council Institute for Laboratory Animal Research *Guide for the Care and Use of Laboratory Animals* and the US Department of Agriculture Animal Welfare Act and Animal Welfare Regulations. Briefly, timed pregnant dams were received from Jackson Laboratories and whole brains were removed and plated into Hank’s (Life Technologies) solution for dissection (10uM of MgCl_2_; 7uM of HEPES; 2 mM glutamine; 100ug/mL penicillin, 100U/mL streptomycin were also added). Cortex was then cut and dissociated by a 10-minute trypsin treatment. Then, 500,000 cortical cells were placed on 6-well Poly-D-Lysine-coated tissue culture plates and maintained in serum-free medium (medium component: neurobasal medium (Life Technologies) containing 1X B27 supplement (Life Technologies), 2 mM glutamine, 100ug/mL penicillin, 100U/mL streptomycin).

Plate-randomized, quadruplicate cortical cultures were transduced at 2 days *in vitro* (DIV2) at an optimized MOI of 3.0. Lentiviral particles remained for 6 hours, after which, particles were removed and replaced with conditioned complete neurobasal medium. Cultures were allowed to mature for an additional 14 days post transduction (DIV16), at which time, total RNA was isolated (described below).

### Hairpin validation

**Figure 1:**
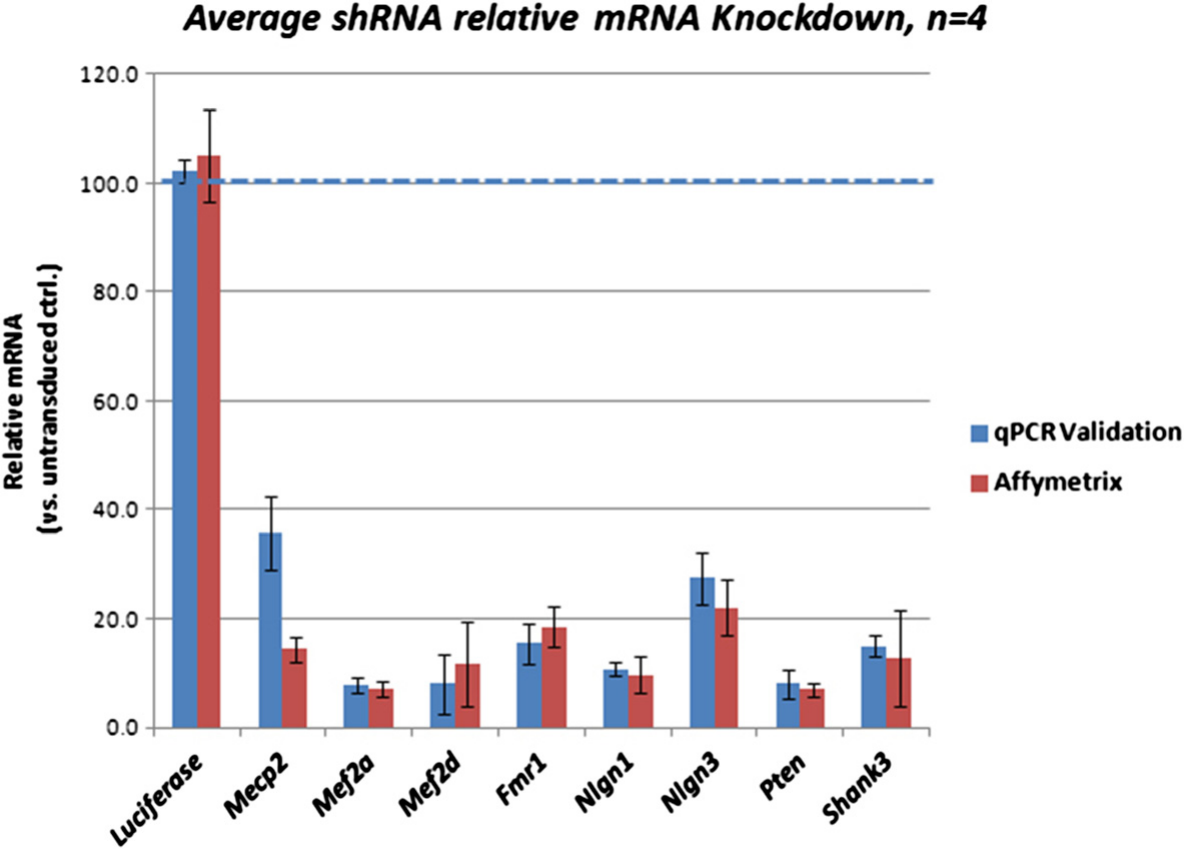
**shRNA efficiently knocks down RNA levels of target genes.** The bars representing the Luciferase-targeting control short-hairpin (sh)RNA-treated neuronal samples are the average values of all target genes. There was no significant difference between the untreated cells and Luciferase shRNA-treated cells for any of the targeted genes.

For each gene target, five unique shRNA targeting lentiviral constructs were generated as described above, along with an shRNA control (designed against luciferase), and used to produce small-scale lentiviral stocks. Viral stocks were used to transduce primary cortical neuronal cultures (see primary neuronal cultures and transduction methods) on DIV2 and cells were grown in culture an additional 7 to 10 days. Total RNA and protein were isolated from replicate cultures. Quantitative PCR (qPCR) (Figure 1) and western blot (Additional file 1: Figure S1) was performed to validate a minimum knockdown level of 75% at the mRNA (described below) and protein levels for all hairpin constructs used in study. *Glyceraldehyde 3-phosphate dehydrogenase* (*Gapdh*) levels were monitored at both the RNA and protein levels as a control. The best-performing hairpin for each gene was carried forward for genome-wide expression analysis.

### RNA isolation, cDNA synthesis and qPCR

Total RNA was isolated utilizing the Qiagen (Germantown, MD, USA) RNeasy mini total RNA isolation kit according to manufacturer’s instructions. RNA quality was validated utilizing a NanoDrop spectrophotometer (Thermo Fisher Scientific, Waltham, MA, USA), assuring at least a 2.0 260/280 ratio was obtained. cDNA was generated from 1 ug total RNA using the Life Technologies High Capacity RNA-to-cDNA kit (number 4390716) according to manufacturer’s instructions. Prior to the Affymetrix (Santa Clara, CA, USA) Gene Chip analysis, qPCR for the target gene was performed on quadruplicate replicates 15 ngs RNA-equivalent cDNA to ensure knockdown. Only samples showing acceptable knockdown (>75% knockdown by mRNA) were submitted for gene chip analysis.

### Microarray hybridization and quality control analysis

Total RNA was hybridized to Affymetrix Mouse 430_2 microarrays at Gene Logic (Gaithersburg, MD, USA). RNA degradation plots were analyzed for quality control purposes. Four samples did not pass quality control (QC) and were omitted from further analysis (one each from the *Mef2d*, *Nlgn1*, *Shank3*, and non-transduced groups). The raw data files were then normalized using robust multi-array averaging (RMA) [40]. Hierarchical clustering by positive correlation (Ward linkage) was performed in Genedata Expressionist. Prior to statistical analysis, probe sets with _x designations were excluded for potential lack of specificity. Additional probe sets were excluded if absolute expression was <50 for all samples (expression was considered absent), resulting in 24,343 probe sets for statistical analysis. Gene expression for all sample types was analyzed on the log2 scale. Linear models were used to calculate *P*-values between the groups of interest. The linear model *t*-statistics were regularized using the moderated-*t* approach of Smyth [41]. Adjustment of *P*-values was performed according to Benjamini and Hochberg [42] to control for multiplicity of testing. Each set of microarrays from a shRNA experiment treatment group were compared to the set of microarrays from the luciferase shRNA control set. Probe sets with false discovery rate (FDR)-corrected *P*-value ≤0.05 and ≥1.5-fold change were identified for each treatment group for pathway analysis, as the historic RT-PCR confirmation rate of microarray data fitting these criteria is approximately 70% (Additional file 2: Table S1). Overlap with a recently published autism gene interactome [20] was performed for all treatment groups. All primary microarray data from this experiment are available in the Gene Expression Omnibus [GEO:GSE47150].

### Bioinformatics analysis of gene expression data

Analyses of gene lists from the miRNA experiments were performed using either Nextbio™ software (Santa Clara, CA, USA, http://www.nextbio.com), Gene Sensor Suite (GSS), or the causal reasoning engine (CRE). The NextBio software uses a modified form of the gene set enrichment algorithm to identify important pathways and other ontologies [43]. All analyses done with NextBio were done utilizing the default parameters. NextBio pathway analysis utilized the pathways compiled by the Broad Institutes gene set enrichment analysis (GSEA) application as part of their molecular signatures database, MSigDB [44]. Related tissues were identified from NextBio’s transcriptional profiles for over 6,000 publically available studies. The GSS application identifies significantly enriched pathways using Fisher’s exact test and corrected for multiple testing using Q-value [45,46]. GSS pathways were generated from Ingenuity pathways from October 2010 (Ingenuity® Systems (Redwood City, CA, USA), http://www.ingenuity.com). The CRE algorithm uses multiple statistical parameters to assess the similarity to upstream effectors and their downstream responses to interpret measured gene expression changes [47,48]. Simply stated, the CRE can be thought of as an enhanced type of gene set enrichment analysis (GSEA). Causal statements were curated from the biomedical literature (Ingenuity and Selventa knowledge bases) in the form of, X (increases or decreases) Y, such that X and Y are measured biological quantities. These quantities can consist of multiple types, including protein modifications, mRNA levels, biological processes and/or chemical compound treatments. The combined knowledge base is then interrogated with the microarray transcriptomic data to infer upstream events (called hypotheses). The CRE algorithm generates statistical stringency by employing two primary methods. First, enrichment of all possible transcripts for the hypothesis is measured, a method shared in common with GSS and GSEA. Second, the method of correctness of the hypothesis is calculated, and is simply the difference of having the correct direction minus the incorrect transcripts observed. There are two advantages in using these methods in the CRE. The first advantage is that it is a specific molecular interaction of the hypothesis that is being evaluated. Second, the directionality of the interaction within the hypothesis is retained by using the correctness parameter. *P*-values were generated and cutoffs were applied using the following filters: correctness *P*-value <0.05, enrichment *P*-value <0.05, minimum number of correctly explained gene expression changes ≥3, percent correctly explained gene expression changes ≥60%, ranking score <100. The hypotheses were deciphered and visualized using the Causal Reasoning Browser, a Java-based plugin for the open source biomolecular interaction viewer Cytoscape (http://www.cytoscape.org)[49].

### BDNF quantitation

Neurons were treated in 24-well plates with blank media or shRNA against luciferase, *Fmr1*, or *Mecp2* in randomized wells across two plates (n = 12 per condition). For protein analysis, neurons were lysed in 20 mM TrisHCl (pH 7), 137 mM NaCl, 1% NP40, 10% glycerol, 1 mM PMSF, 10 μg/mL aprotinin, 1 μg/mL leupeptin, and 0.5 mM sodium orthovanadate. Lysates were centrifuged at 14,000 × g for 30 minutes at 4°C. Supernatants were stored at −80°C until assay. BDNF levels were measured using a modified version of the Promega (Madison, WI, USA) BDNF Emax® Immunoassay system (G7611). Half-volume 96-well ELISA plates (Costar®; Corning, Lowell, MA, USA) were coated with 50 μl anti-BDNF mAb at 1:1000 dilution in 0.025 M sodium bicarbonate and 0.025 M sodium carbonate, sealed, and stored at 4°C overnight. Plates were washed four times with PBS containing 0.05% Tween20, then blocked for 2 hours at room temperature with 130 μl/well Promega blocking buffer (G3311). Samples and standards were prepared in blocking buffer (1:4 dilution), then loaded onto the plates (50 μl) following a wash step. Plates were sealed and stored at 4 °C. On the third day plates were washed and incubated with 50 μl/well anti-human BDNF pAb at 1:500 dilution in blocking buffer for 2 hours at room temperature. Plates were washed again and incubated with 50 μl anti-IgY horseradish peroxidase conjugate at 1:200 dilution in blocking buffer for 1 hour at room temperature. Following a final wash, 50 μl TMB solution was added to each well. The reaction was stopped with 1 N HCl after 10 minutes, and 450 nm optical densities were read on a Spectramax plate reader (Molecular Devices, Sunnyvale, CA, USA). Samples were interpolated off of a standard curve fit by a fourth order polynomial equation. Interpolated BDNF levels were normalized to total protein (DC Protein Assay Kit II, Bio-Rad, Hercules, CA, USA). GraphPad Prism 5.0 was used to perform the Kruskal-Wallis test followed by the Dunn test for multiple comparisons, to determine statistically significant changes (*P*-value <0.05).

## Results

### Confirmation of knockdown

Prior to transcriptomic analysis, individual RNA samples were confirmed for relative knockdown by quantitative RT-PCR. Average knockdown of replicate samples (Figure 1) for the candidate genes were as follows: *Mecp2* (86%), *Mef2a* (94%), *Mef2d* (89%), *Fmr1* (86%), *Nlgn1* (89%), *Nlgn3* (89%), *Pten* (95%) and *Shank3* (90%). Knockdown was normalized to a single untransduced cortical neuronal sample (detected message levels were relative to *Gapdh*). All individual samples showed at least a 75% knockdown of target gene expression providing high confidence that the pathways under investigation were being significantly perturbed. Additional experiments (Additional file 1: Figure S1) indicated that protein levels for all gene products were decreased in conjunction with lentiviral-mediated RNA knockdown in the primary neurons.

### Evaluation of differentially expressed genes

**Figure 2:**
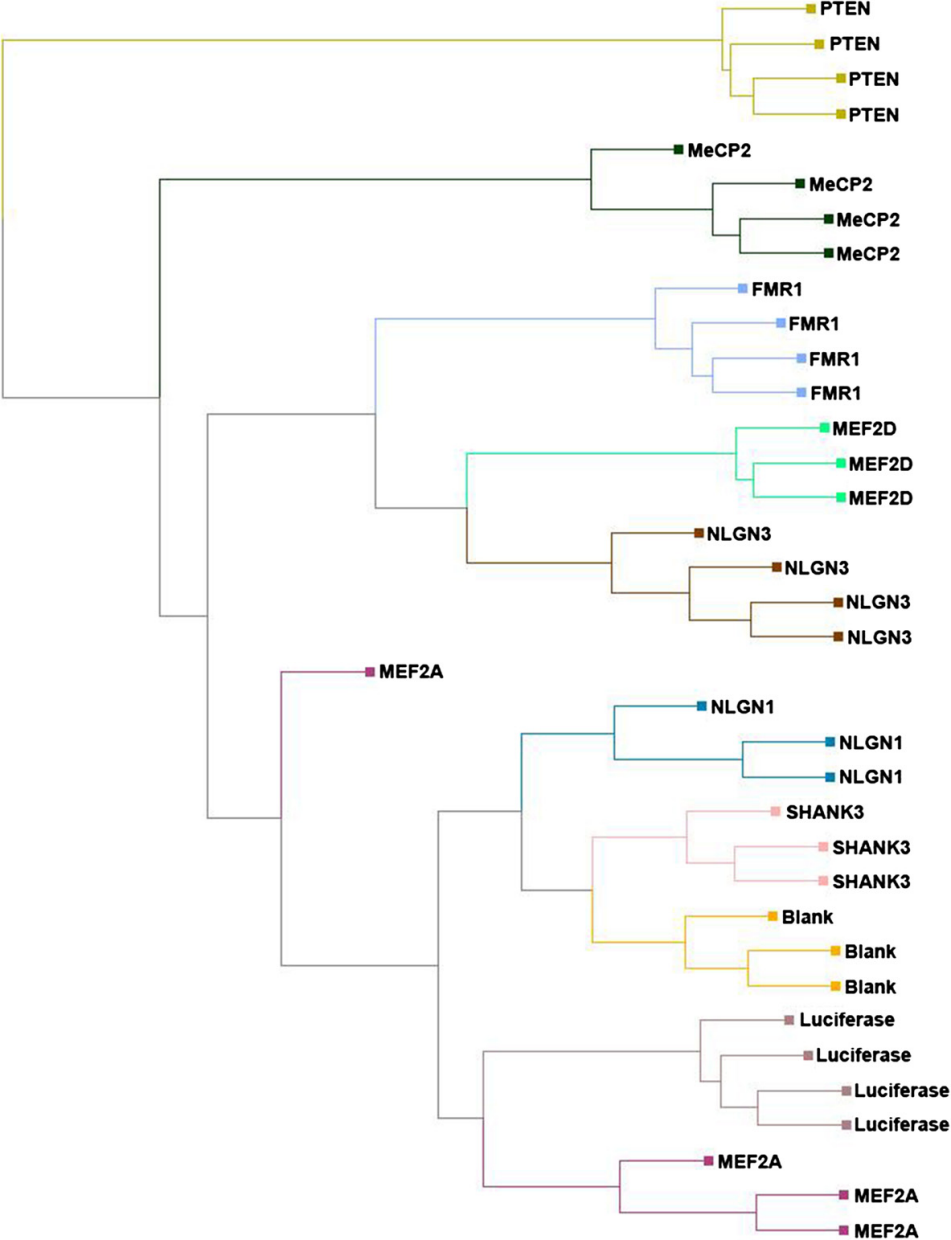
Hierachical clustering of intensity values from individual short-hairpin (sh)RNA knockdown experiments.

Expression values for each of the shRNA-targeted genes as determined by the Affymetrix GeneChips correlated well with values determined by RT-PCR (Figure 1 - Affymetrix data from shRNA-treated samples were compared to the untransfected control for purposes of the figure). Hierarchical clustering of normalized data revealed tight correlation among biological replicates, with the exception of *Mef2a*, in which one sample was separated from the rest (Figure 2). *Pten* was the most distinct treatment group, lying in its own branch of the tree. The next most isolated treatment group was with *Mecp2* knockdown (Additional file 3: Table S2). These treatments produced the most numerous changes in gene expression amongst all the hairpins. The total number of probe sets identified as significantly different from the luciferase control (>1.5 fold at *P* <0.05) in each condition were as follows: *Fmr1* (2,395), *Mef2d* (2,736), *Mef2a* (1,059), *Mecp2* (3,967), *Nlgn1* (1,230), *Nlgn3* (2,224), *Pten* (3,653), and *Shank3* (1,445). Comparison of the luciferase shRNA versus the untransduced control revealed the smallest number of significant changes - 997. As an early determination of the relevance of cell-culture knockdown to the known molecular biology of ASD, the current datasets were evaluated for enrichment in an ASD gene interactome established by Sakai *et al*. [20]. Although the luciferase versus blank condition was not significantly enriched for genes in this interactome, *Fmr1*, *Mecp2*, *Mef2a*, *Mef2d*, *Nlgn1*, *Pten*, and *Shank3* shRNA transcriptomes all showed significant overlap (Table 3). The most frequently identified ASD interactome gene was *CAMK2A*, which was upregulated by *Fmr1* shRNA, but downregulated by all of the other ASD gene shRNA targets.

**Table 3:** Autism spectrum disorder interactome genes significantly altered by three or more shRNA treatments

**Gene**	**Gene description**	**Fold-change**							
		**Fmr1**	**Mecp2**	**Mef2a**	**Mef2d**	**Nlgn1**	**Nlgn3**	**Pten**	**Shank3**
*Camk2a*	Calcium/calmodulin-dependent protein kinase II alpha	1.69	−2.00	−1.81	−1.65	−1.85	−1.68	−2.53	−1.68
*Prmt1*	Protein arginine N-methyltransferase 1	1.55	2.32		2.02	1.65	2.17	1.79	2.06
*Cacna1c*	Calcium channel, voltage-dependent, L-type, alpha 1C subunit	1.68	1.75	1.69	1.90	1.64	1.67	2.23	
*Sh3gl3*	SH3-domain GRB2-like 3		1.59	1.70	1.62	1.59	1.63	1.84	1.56
*R3hdm1*	R3H domain 1 (binds single-stranded nucleic acids)	2.40	−2.09	−1.63	1.92		−1.65	−1.88	
*Tgfb1i1*	Transforming growth factor beta 1-induced transcript 1				−2.96	1.74	−3.19	2.48	
*Lims2*	LIM and senescent cell antigen-like domains 2		2.43		1.75			3.80	1.90
*Kif17*	Kinesin family member 17	−2.01	−2.13		−2.36		−1.70		
*Nkd2*	Naked cuticle 2 homolog (Drosophila)	−1.78	−2.12		−1.83		−1.77		
*Capn3*	Calpain 3		−1.65	1.53	2.98			−1.77	
*Nell1*	NEL-like 1 (chicken)	2.23	−1.85		−1.58	1.64			
*PYCR1*	Pyrroline-5-carboxylate reductase 1	−1.56	−2.81				−2.00	1.55	
*TBC1D9B*	TBC1 domain family, member 9B (with GRAM domain)	1.94	−2.08	1.57				1.50	
*Prr13*	Proline-rich 13	−1.81	−1.77		−1.71		−1.82		
*STK32C*	Serine/threonine kinase 32C		−1.89				−1.64	−1.69	−1.51
*DLGAP2*	Discs, large (Drosophila) homolog-associated protein 2	1.80		−1.60		−2.10			
*SOD1*	Superoxide dismutase 1, soluble			−1.64				−2.22	−1.69
*Dlgap1*	Discs, large (Drosophila) homolog-associated protein 1	1.91			−2.45			−1.86	
*MBD2*	Methyl-CpG binding domain protein 2			−1.94			−1.58		−1.55
*FBLN5*	Fibulin 5	1.52	2.15					2.69	
*Sertad1*	SERTA domain-containing 1				−1.67		−1.95		−1.69
*Bhlhe40*	Basic helix-loop-helix family, member e40		−2.28		−1.85		−1.74		
*Slit2*	Slit homolog 2 (Drosophila)		−1.68		−2.43			−2.09	
*Actn2*	Actinin alpha 2		−2.64		−1.53		−1.65		
*Ube3a*	Ubiquitin protein ligase E3A		−2.48		−1.52				−1.53
*Slc13a3*	Solute carrier family 13 (sodium-dependent dicarboxylate transporter), member 3				2.32		1.56	−1.60	
*NOMO1*	NODAL modulator 1			1.64				1.53	1.54
*RALBP1*	RalA binding protein 1	1.65		1.52				1.73	
*MYH10*	Myosin, heavy chain 10, non-muscle	−2.12	−1.78					−1.53	
*HMG20A*	High-mobility group 20A		1.68	1.59				1.76	
*BAIAP2*	BAI1-associated protein 2					−1.51	−1.73	−1.55	
*Fxr1*	Fragile X mental retardation gene 1, autosomal homolog	1.61	1.56		1.99				
*Lingo1*	Leucine-rich repeat and Ig domain-containing 1				−1.56			−1.73	−1.57
*Nup62*	Nucleoporin 62	−1.54			−1.59		−1.70		
*Notch2*	Notch gene homolog 2 (Drosophila)		1.69		1.52			−2.06	
*EML1*	Echinoderm microtubule-associated protein-like 1	−1.64	1.72					−1.72	
*NECAB3*	N-terminal EF-hand calcium-binding protein 3		−1.79				−1.52	−1.75	
*Tcf25*	Transcription factor 25 (basic helix-loop-helix)				−1.51		−1.69	−1.54	

### NextBio detection of related transcriptional profiles

The Nextbio database allows for comparison of transcriptional profiles between datasets and transcriptional profiles for over 6,000 publically available studies. The most highly correlated datasets for any of the ASD gene shRNA profiles were other ASD shRNA profiles from this experiment. As a control, the *Mef2a* and *Mef2d* profiles were compared against a published study in which the same hippocampal neurons were transduced with both *Mef2a* and *Mef2d*[32]. The published study showed significant positive correlation with the present *Mef2a* and *Mef2d* datasets, with 107 genes in common with *Mef2a* profile and 283 genes in common with the *Mef2d*. Similarly, a comparison with microarray analysis of cortex from *Mecp2* knockout mice showed significant overlap with this *Mecp2* shRNA transcriptional profile (445 genes in common, *P* = 1 × 10^-8^) [6]. The most highly correlated publically available transcriptional profiles for the remaining ASD-related genes came from comparisons of mouse brains at various postnatal ages to embryonic day 14.5 [50] or up to birth [51]. A time course of primary mouse hippocampal neurons *in vitro*[52] was also correlated with all shRNA treatments (data not shown). All of these developmental datasets showed significant inverse correlation with all shRNA treatments, including luciferase. Given the number of activity-dependent genes affected by shRNA treatment, the correlations with developmental datasets suggest that lentiviral gene delivery may have nonspecifically altered the development of the mouse neurons. Therefore, care was taken to subtract any changes observed in the lentiviral-treated cells from the other datasets for all analyses in an attempt to minimize the impact of this potential artifact.

### Pathway analysis

NextBio analysis of the MSigDB pathways yielded a large number of canonical pathways significantly enriched in one or more treatment group. Three of these pathways were significantly enriched in the luciferase versus blank comparison but not in any other dataset. In addition, the luciferase versus blank dataset yielded a number of pathways in common with other shRNA datasets. These pathways were considered nonspecific and excluded from further analysis. After excluding these pathways, 256 canonical pathways were significantly enriched in one or more treatment groups. Many of these pathways were affected by more than one condition; 26 pathways were significantly enriched by 5 or more ASD shRNA datasets and the top 15 most conserved are shown in Table 4. The most frequently enriched pathway was the *Neurotrophic tyrosine kinase receptor type 1(TrkA)* receptor pathway, in which all datasets had a significant number of downregulated genes except *Pten*, which had a significant number of upregulated genes. Other pathways affected by multiple ASD gene shRNA targets included signaling pathways related to additional genes implicated in ASD, such as *Neuregulin*, *Mammalian Target Of Rapamycin* (*mTOR*), and *Reelin*.

**Table 4:** Canonical pathways in NextBio that were significantly enriched following ASD gene-targeted shRNA constructs, but not affected by luciferase shRNA

	**ASD gene count**	**Fmr1**		**Mecp2**		**Mef2a**		**Mef2d**		**Nlgn1**		**Nlgn3**		**Pten**		**Shank3**	
**Biogroup**		**Up**	**Down**	**Up**	**Down**	**Up**	**Down**	**Up**	**down**	**Up**	**Down**	**Up**	**Down**	**Up**	**Down**	**Up**	**Down**
TrkA receptor	8		2		4		2		2		2		3	3			2
Erythropoietin-mediated neuroprotection through NFKB	7	3	2		3		3		1		2		1	3			
PGC1A pathway	7				8		5		7		3		4		9		4
Long-term potentiation	7	8			18		5		15	3					16		6
Cell adhesion molecules (CAMs)	7			10	14		8		13		8		11	13	12		10
G2 and M phases	7		1		1		1	1	1		1		2	3			
P53 HYPOXIA pathway	7		3		3		3		3		2		3	6			
Biosynthesis of steroids	6		8		5				3	2			5		4		
Arginine and proline metabolism	6		8		9	1			8				8	5			
Antigen processing and presentation	6				9		3		6		3		10				4
BAD pathway	6		3				3		4	2		2					2
Neuroregulin receptor degredation protein-1 controls ERBB3 receptor recycling	6			1		1		1		1		1				1	
Eicosanoid synthesis	6						1		1		1		1	3			2
Phosphatidylinositol signaling system	5	10			17						6		11		10		
Vitamin B6 metabolism	5	1			3			1	1			1	2		1		

ASD gene count, number of conditions in which the pathway was significantly affected (that is, the number of different shRNAs that altered that particular pathway); Up, numbers of member genes altered in each pathway at least 1.5-fold broken out by positive fold-changes; Down, numbers of member genes altered in each pathway at least 1.5-fold broken out by negative fold-changes; ASD, autism spectrum disorder; NFKB, Nuclear factor KB; TrkA, Neurotrophic tyrosine kinase receptor type 1; PGC1A, Peroxisome proliferator-activated receptor gamma coactivator 1-A ERBB3, Receptor tyrosine-protein kinase erbB-3.

GenSensor analysis of the Ingenuity pathways resulted in 114 canonical pathways which were significantly over-represented in one or more of the ASD shRNA treatment groups. The top fifteen most conserved pathways are show in Table 5 (see Additional file 4: Table S3 for the complete list). In the pathways found only in the ASD shRNA treated samples, there are a number of pathways related to neuronal signaling, in particular to cyclic AMP signaling. Thirteen of these pathways were significantly enriched in the luciferase versus blank comparison (yellow highlighted). These pathways may indicate general effects of the shRNA delivery system on the neuron cell culture. However, because GSS does not consider the magnitude of gene expression change, these results do not preclude real treatment-related effects on these pathways above the background levels induced by the lentivirus. Only two pathways were found to be enriched solely in the luciferase versus blank comparison.

**Table 5:** Ingenuity pathways that were significantly enriched in multiple ASD short-hairpin (sh)RNA knockdown experiments

**Description**	**Blank pathway ranking**	**Fmr1 pathway ranking**	**Mef2a pathway ranking**	**Mef2d pathway ranking**	**Mecp2 pathway ranking**	**Nlgn1 pathway ranking**	**Nlgn3 pathway ranking**	**Pten pathway ranking**	**Shank3 pathway ranking**	**Pathway count**	**Average ranking**
*Axonal guidance signaling*	*4*	*1*	*16*	*2*	*1*	*2*	*1*	*4*	*1*	*8*	*3.50*
*Molecular mechanisms of cancer*	*6*	*5*	*2*	*11*	*6*	*1*	*2*	*1*	*6*	*8*	*4.25*
*Breast cancer regulation by stathmin1*	*13*	*8*	*1*	*8*	*4*	*3*	*5*	*2*		*7*	*4.43*
*G-protein coupled receptor signaling*		*2*	*10*	*5*	*8*			*9*		*5*	*6.80*
Protein kinase A signaling		6		18	2		14	3		5	8.60
Huntington’s disease signaling			23	1	37			22	3	5	17.20
*Corticotropin-releasing hormone signaling*			*4*	*9*	*31*		*7*	*36*		*5*	*17.40*
Calcium signaling	8		15	12	44			43	11	5	25.00
Amyotrophic lateral sclerosis signaling		4		15	66			41	7	5	26.60
Synaptic long-term potentiation			5	6	5			6		4	5.50
cAMP-mediated signaling			8	7	3			7		4	6.25
GNRH signaling			6	20	9			13		4	12.00
*Neuropathic pain signaling in dorsal horn neurons*		*7*	*21*	*3*				*18*		*4*	*12.25*
Neuroprotective role of THOP1 in Alzheimer’s disease	7	23	19			4	6			4	13.00
Role of NFAT in cardiac hypertrophy				17	7		9	19		4	13.00

Numbers are odds ranking of the pathways in each shRNA experiment. All pathways were sorted by number of shRNA experiments in which they appear (high to low) followed by average ranking in experiments (low to high). Italicized pathways were also enriched in the luciferase control experiment. GNRH, gonadotrophin-releasing hormone; THOP, Thimet oligopeptidase 1; NFAT, Nuclear factor of activated T-cells.

### Causal reasoning engine - molecular network inferences

**Figure 3:**
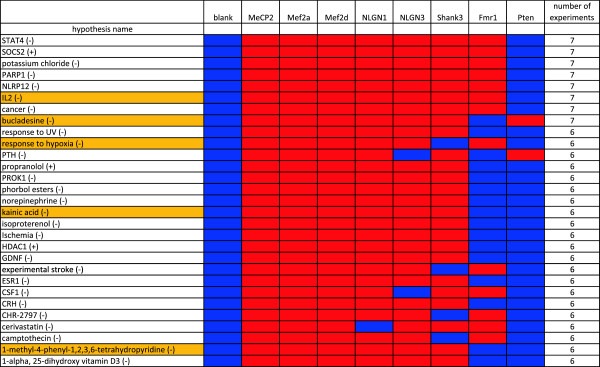
**Diverse set of ASD-associated genes produce similar pathway-level perturbations when knocked down.** A total of 269 hypotheses were observed in at least three of the experiments and not in the blank. Only the hypotheses that were observed in at least 6 of the treatment conditions are included in the figure (red squares indicate that the hypothesis was identified for that experimental condition). Additional file 5: Table S4 list contains a full list of the 269 hypotheses. The notation (+) indicates the hypothesis is predicted to be upregulated, and (−) indicates it is predicted to be downregulated. Names highlighted in orange are part of the molecular interaction network (Figure 4).

**Figure 4:**
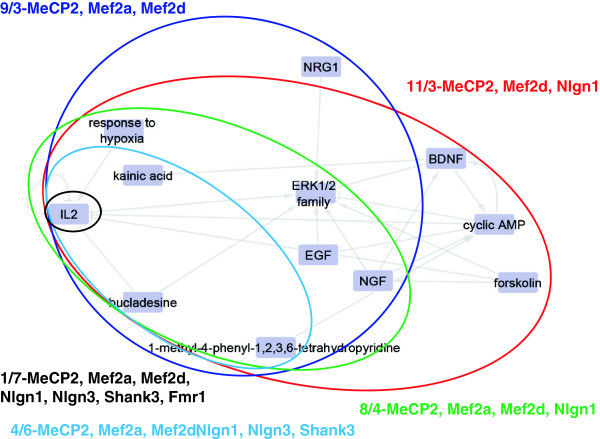
**Multiple causal reasoning engine (CRE)-predicted hypotheses collapse into a molecular interaction network.** The edges of the connections represent the information linking the nodes, for example, a PubMed reference for the interaction. The hypotheses are colored based on the direction of regulation: blue nodes are predicted down, whereas yellow are predicted up. Circled regions indicate significant hypotheses seen in different short-hairpin (sh)RNA experiments: red - *Myocyte enhancer factor* (*Mef*)*2d*, *Methyl-CpG binding protein* (*Mecp*)-*2*, *Neuroligin* (*Nlgn1*); blue - *Mef2a, Mef2d*, *Mecp2*; green - *Mef2a Mef2d*, *Mecp2*, *Ngln1*; light blue - *Mecp2*, *Mef2a*, *Mef2d*, *Nlgn1*, *Nlgn3*, *SH3 and multiple ankyrin repeat domains* (*Shank*)*3;* black - *Mecp2*, *Mef2a*, *Mef2d*, *Nlgn1*, *Nlgn3*, *Shank3*, *Fragile X mental retardation* (*Fmr*)*1* (IL2-hypothesis).

CRE analysis yielded a large number of causal hypotheses for each ASD shRNA treatment group. The rank order for the hypotheses was as follows: *Mecp2* (218) >*Mef2d* (216) >*Nlgn3* (174) >*Mef2a* (170) >*Nlgn1* (151) >*Shank3* (82) >*Fmr1* (45) >*Pten* (12). The top fifteen hypotheses shared between the highest number of experiments is shown in Figure 3 (See Additional file 5: Table S4 for the complete list). There was no hypothesis that was seen in all eight experimental conditions and not in the blank. Looking at the experiments broadly, two groups emerge based on the conservation. *Mecp2*, *Mef2d*, *Nlgn3*, *Mef2a*, *Nlgn1* and *Shank3* share similar hypotheses and were more dynamic, generating 3 to 5 times more hypotheses. In contrast, the same hypotheses are not seen being implicated for the *Fmr1* and *Pten* experiments, with the latter experiment appearing quite different than the rest. The predicted hypotheses are overwhelmingly downregulated, with 86% (230 of 268) with the respective order of contribution being 87% (*Mecp2*), 89% (*Mef2d*), 84% (*Nlgn3*), 86% (*Mef2a*), 87% (*Mef2a*), 76% (*Nlgn1*), and 79% (*Fmr1*); *Pten* again is the exception with only 58%. Figure 4 is a composite of the most conserved hypotheses generated by CRE for the seven concordant treatment groups. Recurring hypotheses are highlighted with circles. The central hubs of the network are cyclic AMP and the extracellular signal-regulated kinase (ERK)1/2 family, which are directly connected to seven and eight primary hypotheses respectively.

### Confirmation of BDNF protein response

**Figure 5:**
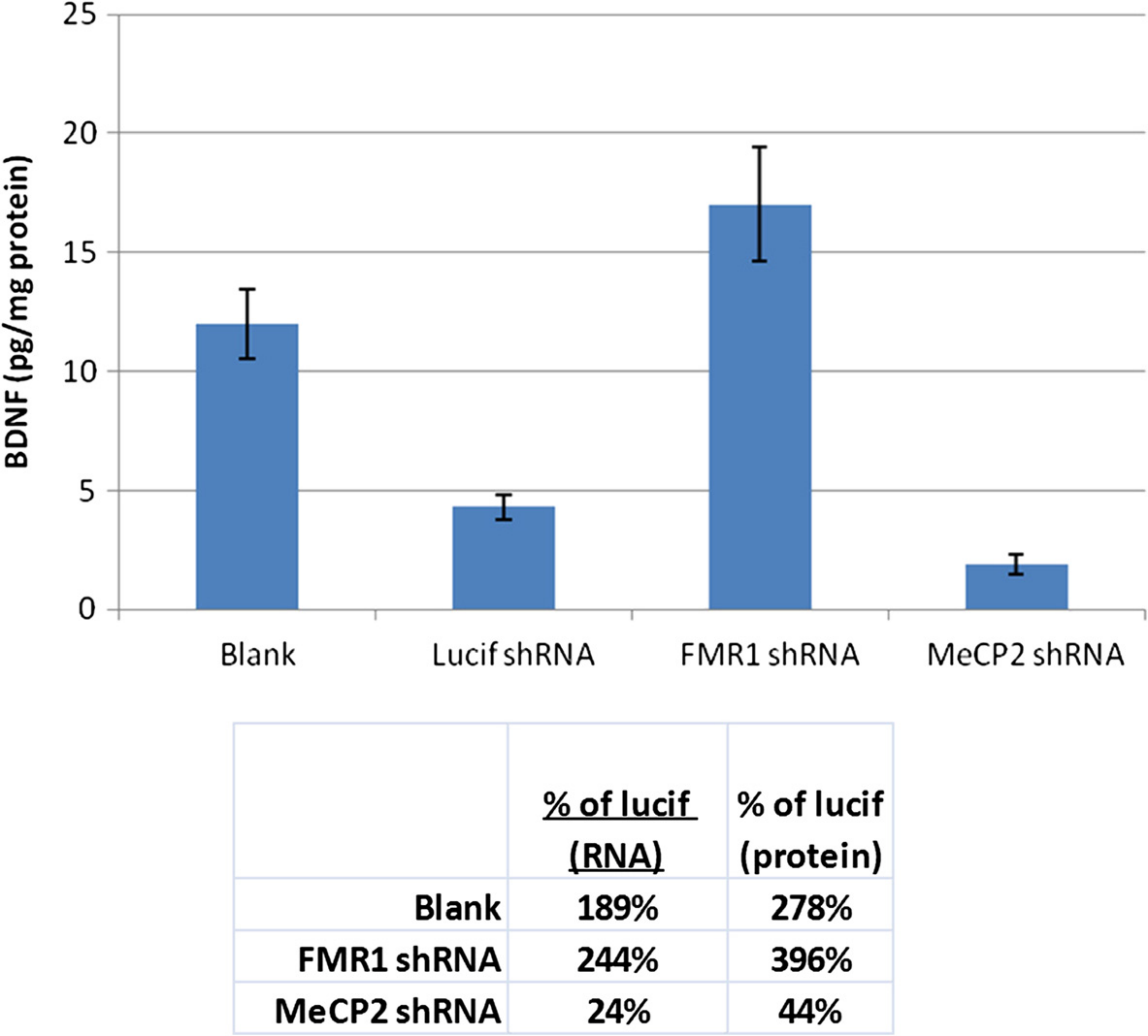
**Measurement of BDNF protein levels in 14-days-*in vitro *mouse cortical neurons following transduction with short-hairpin (sh)RNA constructs for luciferase (non-mammalian control), *Fragile X mental retardation*(*Fmr1*), *Methyl-CpG binding protein*(*Mecp2*) or non-transduced neurons (blank).** Values for mRNA and protein as % of luciferase control shRNA are shown in the table on the right (n = 12 per group).

Given that multiple hypotheses predicted from multiple target-knockdown datasets converge on *Bdnf*, regulation of *Bdnf* could play a central role in ASD pathobiology. In order to confirm that these predicted changes in BDNF were accurate and that the transcriptional changes measured translated to the protein level, the two shRNA conditions in which *Bdnf* mRNA was most robustly altered (*Fmr1* and *Mecp2*) were evaluated for impacts on BDNF protein. Neurons were treated in the same manner as for the microarray study, and lysates were harvested and analyzed by ELISA for BDNF. As predicted by mRNA levels, the luciferase shRNA construct alone significantly lowered BDNF (*P*-value <0.05) (Figure 5). *Mecp2* shRNA further reduced BDNF levels, while *Fmr1* shRNA significantly increased BDNF levels relative to luciferase shRNA (*P*-value <0.05).

## Discussion

ASD is a neurological disorder with a strong genetic component that has been linked to a number of gene defects. These genes have a broad range of activities, ranging from membrane receptors and scaffold proteins to metabolic regulators and transcription factors [25,35]. Despite this diversity, ASD patients manifest with similar behavioral and neuronal phenotypes, albeit with different severities. This commonality of neurological phenotype suggests that the genetic defects may act through a limited set of pathways. In this report, we employed shRNA knockdown of eight ASD relevant genes in neuronal culture to explore the downstream effects and identify common pathways or transcriptional signatures.

Following microarray analysis of all samples, we performed cluster analysis on the intensity values. As expected, samples clustered by treatment group, demonstrating an overall consistency and quality of the knockdown experiment and subsequent gene expression quantitation. It also illustrates the distinctiveness of the downstream expression effects of knockdown of individual genes. Knockdown of *Pten* and *Mecp2* had the most dramatic effects on gene expression. Given *Pten*’s broad role in numerous cellular processes and *Mecp2*’s role as a transcription factor, these results were not unexpected. For example, mutations in *Pten* have been linked not only to ASD but also cancer and diabetes [53,54]. Fragile X mental retardation protein (FMRP), the protein product of *Fmr1*, has been shown to interact with a larger number of target proteins in relation to dendritic control of translation. A list of FMRP target proteins showed significant enrichment in the transcriptional profiles of shRNA for not only *Fmr1*, but also *Mecp2*, *Pten*, *Shank3*, *Nlgn1* and *Nlgn3*[25]. We further compared the genes affected in one or more knockdown experiments to a list of ASD interactome genes [20]. This comparison indicated that knockdown of the eight ASD genes resulted in changes to a significant number of ASD interactome genes and the genes affected by the luciferase shRNA condition had little overlap with the ASD genes (Table 3). This control comparison is important, as other groups have reported nonspecific adverse effects of other shRNA and siRNA constructs [55,56]. The luciferase shRNA versus untransduced comparison yielded almost 1,000 differentially expressed transcripts, with an impact on BDNF measured at the protein level. Thus, by identifying the changes in the luciferase shRNA versus untransduced experiments and subtracting those, the subsequent pathway analyses could focus on pathways that were specifically targeted by knockdown of the ASD-relevant genes and not identify artifacts of the transduction.

We next analyzed the gene lists from the shRNA experiments by two pathway analysis approaches to obtain different perspectives on the data. The most prominent pathways revealed through analysis with NextBio were a number of pathways related to neurologic signaling and function (Table 4). Secondarily, NextBio indicated that several pathways involved general cellular metabolism and growth were also affected. One prominent pathway, the Peroxisome proliferator-activated receptor gamma coactivator 1-A (PGC1A) pathway, is based on the MSigDB’s version of BioCarta’s pathway and contains *Mef2A* and several calcium-dependent kinases, which show gene expression changes in all shRNA experiments. One aspect of the NextBio analysis is that directionality of change (that is, upregulated or downregulated) is reported. The majority of the pathways are downregulated with ASD shRNA knockdown, suggesting that the genes we chose for this work are needed for the expression of these pathways and thereby their activity. Pathway analysis with GenSensor also identified a number of pathways related to neuronal signaling and function (Table 5). As with the NextBio analysis, several growth and metabolism pathways were also affected.

During an examination of the individual pathways identified by the two pathway analysis methods, we noted a recurring involvement of the mitogen-activated protein kinase kinase (MEK)/ERK signaling pathway. These effects would occur either directly through a kinase signaling cascade (downstream of BDNF/TRK) or via cAMP (as in the case of the dopamine and serotonin G-protein coupled receptors). To further investigate this potential commonality, we employed CRE analysis to identify potential underlying mechanisms (CRE hypotheses) in the shRNA datasets. Unlike pathway analysis, which identifies pathways with altered gene expression, CRE analysis predicts potential mechanisms behind gene changes based on the concordance of the number of genes that change expression, and the directionality of that change [47]. The results of CRE analysis are interlinked hypotheses of potential driving mechanisms or experimental treatments that exhibit similar gene changes. It is interesting that three of the eight most conserved hypotheses have a biological function suggestive of growth and/or immune function, suggesting similar driving mechanisms (Figure 3). Likewise, there are highly conserved hypotheses involved with neurogenesis, synaptic activity and differentiation, as expected, although not mutually exclusive. Choosing the *Mef2d* experiment as a representative of the six most conserved shRNA treatments, the top ranking clusters can be connected as a molecular interaction map with (Figure 4), cyclic AMP and ERK serving as dual hubs of the network directly connecting seven and eight related hypotheses respectively. Three of the experiments, *Mecp2*, *Mef2d*, and *Nlgn1*, shared 11 of the 12 hypotheses in the network. As more experiments are included, the shared number of hypotheses decreases, for example, the light blue grouping of six experiments including *Shank3* is based on four hypotheses.

In addition to this work, other work directly or indirectly supports a role for ERK signaling in the development of ASD. For example, maternal use of one of several different classes of drugs relevant to ERK modulation has been reported to increase the risk of having children born with ASD [57]. Cocaine use during pregnancy has been reported to increase the rate of autism by 11%. Cocaine use has also been shown to alter dopamine-induced phosphorylation of ERK via cAMP [58,59]. Recently, Hoffmann *et al*. showed that chronic cocaine use in rats can lead to attenuated ERK signaling [60]. Chronic maternal cocaine use might thereby attenuate ERK signaling in the fetus. Similarly, mothers taking valproic acid, an inhibitor of gamma-aminobutyric acid (GABA) function, have been demonstrated to have an increased risk of have children with autism [61]. As with cocaine, valproic acid activates ERK signaling [62]. Zou *et al*. demonstrated that RAS/RAF/ERK1/2 signaling was upregulated in the brains of the BTBR mouse model of autism [63]. Recently, the upregulation of this pathway (and of ERK5) has been shown to occur in the brains of autistic subjects [64]. Although misregulation of ERK does appear to be a common feature of ASD, the observed directionality of that misregulation has been contradictory. In the case of Rett syndrome, ERK signaling through the BDNF pathway in particular is reduced. BDNF levels are reduced in *Mecp2*-null mice, and exogenous BDNF has been shown to rescue deficits due to *Mecp2* deficiency [7,9]. In human Rett syndrome patients, a Val/Met polymorphism in BDNF has been associated with disease severity [18]. In the present study, *Mecp2* shRNA produced a significant reduction in *Bdnf* at both the mRNA and protein level, both of which were inversely affected by *Fmr1* knockout. Given the diverse functions of BDNF in neurons, it would be interesting to determine in follow-up studies whether inverse functional outcomes may be observed with these treatments.

Based on the experimental data presented here and previously existing data, we have put together a pathway model to show that the transcriptional regulation exerted by a diverse set of ASD-associated genes converges on ERK signaling. A central role for ERK signaling would explain many of the features associated with ASD. Early work on the ERK proteins described these as microtubule-associated protein 2 kinases, and were shown to phosphorylate MAP2 kinases, proteins known to be involved in neuronal architecture [65-67]. Later work demonstrated that ERK plays a critical role in microtubule formation and thereby to axon/dendrite formation [68,69]. A review article by Hoogenraad and Akhmanova has summarized the criticality of microtubules in synaptic plasticity [70]. Mutations that lead to altered ERK activity would then be expected to have alterations in axon extension and/or retraction and thereby, synaptic plasticity. Mazzucchelli *et al*. found that ERK1-knockout mice exhibit enhanced synaptic plasticity, most likely through the compensatory activation of ERK2 [71]. Voineagu *et al*. recently reported that the expression differences between the temporal and frontal lobes are significantly attenuated in individuals with autism [22]. They further suggested that this lack of differentiation is the mechanism behind the lack of long-range axonal connections and the decreased myelin thickness in autistic prefrontal lobes as reported by Zikopoulos and Barbas [21,22]. In some instances altered ERK activity could interfere with neuroglia wrapping of neuritis to form the myelin sheath. Newbern *et al*. recently reported that ablation of ERK1/2 in Schwann cell precursors resulted in hypomyelination of axons [72].

## Conclusions

A large number of genetic mutations and CNVs have been linked to ASD. The implicated genes span a variety of functions and pathways [25,35]. Despite this diversity, defects in neuronal plasticity and dendrite morphology are commonly associated with this disease. In this report, we utilized shRNA knockdown of eight ASD-associated genes to examine downstream transcriptional alterations and to look for pathway-level commonalities. An underlying assumption is that dysregulation of these genes in primary mouse cortical neurons produce transcriptional alterations robust enough to be detected in lysates of these mixed cultures. As it is difficult in such an experiment to identify a single causal gene, analyzing changes at the pathway level mitigates the reliance on just one or two genes. Pathway analysis by two different approaches both identified alterations in a number of conserved neuronal signaling pathways. Detailed examination of those pathways emphasized alterations to the cAMP and ERK signaling pathways. These pathways would be good starting points for further functional characterization of common downstream neuronal phenotypes following known-down of ASD-associated genes. For example, cAMP reporter assays and phosphoproteomic analysis of ERK pathway regulation would be informative in searching for common intervention points that might reverse the phenotypes caused by the ASD gene disruption. The prospect that multiple genes tied to a single disorder converge on a common set of pathways provides hope that therapeutics can be developed that will be efficacious in a patient population with a heterogeneous genetic background.

## Abbreviations

ASD: Autism spectrum disorder; BDNF: Brain-derived neurotrophic factor; CNV: Copy number variant; CRE: Causal reasoning engine; DIV: Days *in vitro*; ELISA: Enzyme-linked immunosorbent assay; ERBB3: Receptor tyrosine-protein kinase erbB-3; Erk: Extracellular signal-regulated kinase; FDR: False discovery rate; FMRP: Fragile X mental retardation; Gapdh: Glyceraldehyde 3-phosphate dehydrogenase; GFP: Green fluorescent protein; GSEA: Gene set enrichment analysis; GSS: Gene Sensor Suite; GWAS: Genome-wide association studies; MECP2: Methyl-CpG binding protein 2; MEF: Myocyte enhancer factor; MOI: Multiplicity of infection; NLGN: Neuroligin; PBS: Phosphate-buffered saline; PTEN: Phosphatase and tensin homolog; qPCR: quantitative polymerase chain reaction; RT: Reverse transcriptase; SHANK3: SH3 and multiple ankyrin repeat domains 3; shRNA: short-hairpin RNA; TRKA: Neurotrophic tyrosine kinase receptor type 1.

## Competing interests

All authors were employees of Pfizer Global Research and Development, which funded this study, at the time the experimental work was conducted.

## Authors’ contributions

EG carried out the knockdown experiments, including the qPCR analysis and the preparation of samples for transcriptomic analysis. TAL, MMG, and JEF conducted the bioinformatic analysis of the transcriptomic data. TAL, LWF, and DTS conceived of the original project and experimental design. MTP coordinated data analysis and the preparation of the manuscript. EG, TAL, MMG, JEF, and MTP contributed to drafting of the manuscript. All authors read and approved the final manuscript.

## Supplementary Material

Additional file 1: Figure S1 Western blot analysis of protein knockdown. Click here for file

Additional file 2: Table S1 Full gene expression data. Click here for file

Additional file 3: Table S2 Comparison of individual gene expression changes between knockdown datasets. Genes that had fold-changes >1.5 versus control with a *P*-value <0.5 were compared between experiments for overlap. The number of genes found in both knockdown experiments for any combination is shown on the table. Click here for file

Additional file 4: Table S3 Gene set enrichment analysis utilizing Ingenuity pathways. Click here for file

Additional file 5: Table S4 A comparison of common causal reasoning engine (CRE) hypotheses from the nine experiments A total of 269 hypotheses were observed in at least three of the experiments and not in the blank. The notation (+) indicates the hypothesis is predicted to be upregulated, and (−) predicted it to be downregulated. Names highlighted in orange are part of the molecular interaction network (Figure 4). An observed hypothesis (indicated by a red box) satisfies the following filters: correctness *P*-value <0.05, enrichment *P*-value <0.05, minimum number of correctly explained gene expression changes ≥3, percent correctly explained gene expression changes ≥60%, ranking score <100. Click here for file
