# Supplementary figures and images for: Transcriptomic analysis of genetically defined autism candidate genes reveals common mechanisms of action
Source: Mol Autism. 2013 Nov 15;4:45. doi: 10.1186/2040-2392-4-45 (PMC4176301; doi:10.1186/2040-2392-4-45)

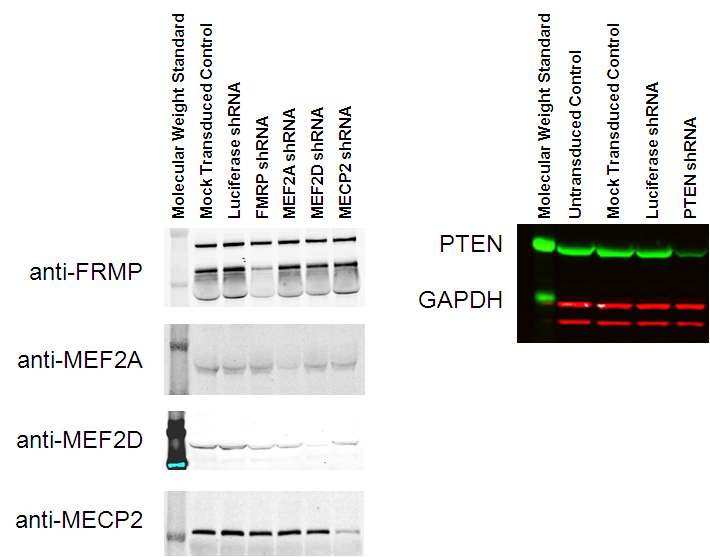

Supplement: Additional file 1: Figure S1 — Western blot analysis of protein knockdown. [file 2040-2392-4-45-S1.jpeg]
