# Supplementary material for: Transcriptomic analysis of genetically defined autism candidate genes reveals common mechanisms of action
Source: Mol Autism. 2013 Nov 15;4:45. doi: 10.1186/2040-2392-4-45 (PMC4176301; doi:10.1186/2040-2392-4-45)
Supplement: Additional file 5: Table S4 — A comparison of common causal reasoning engine (CRE) hypotheses from the nine experiments A total of 269 hypotheses were observed in at least three of the experiments and not in the blank. The notation (+) indicates the hypothesis is predicted to be upregulated, and (−) predicted it to be downregulated. Names highlighted in orange are part of the molecular interaction network (Figure 4). An observed hypothesis (indicated by a red box) satisfies the following filters: correctness P-value <0.05, enrichment P-value <0.05, minimum number of correctly explained gene expression changes ≥3, percent correctly explained gene expression changes ≥60%, ranking score <100. [file 2040-2392-4-45-S5.pdf]

Supplementary table 4 - Hypotheses common in ≥ 3 experiments

	blank	MeCP2	Mef2a	Mef2d	NLGN1	NLGN3	Shank3	Fmr1	Pten	number of experiments
hypothesis name										
STAT4 (-)										7
SOCS2 (+)										7
potassium chloride (-)										7
PARP1 (-)										7
NLRP12 (-)										7
IL2 (-)										7
cancer (-)										7
bucladesine (-)										7
response to UV (-)										6
response to hypoxia (-)										6
PTH (-)										6
propranolol (+)										6
PROK1 (-)										6
phorbol esters (-)										6
norepinephrine (-)										6
kainic acid (-)										6
isoproterenol (-)										6
Ischemia (-)										6
HDAC1 (+)										6
GDNF (-)										6
experimental stroke (-)										6
ESR1 (-)										6
CSF1 (-)										6
CRH (-)										6
CHR-2797 (-)										6
cerivastatin (-)										6
camptothecin (-)										6
1-methyl-4-phenyl-1,2,3,6-tetrahydropyridine (-)										6
1-alpha, 25-dihydroxy vitamin D3 (-)										6
U0126 (+)										5
tributyrin (-)										5
TP53 (-)										5
T113242 (-)										5
STAT3 (-)										5
SMAD3 (-)										5
SB203580 (+)										5
RHO (+)										5
Response to stress (-)										5
poly rI:rC-RNA (-)										5
Pkc family (-)										5
PGR (-)										5
paclitaxel (-)										5
okadaic acid (-)										5
NGF (-)										5
N-Ac-Leu-Leu-norleucinal (+)										5
N-acetylsphingosine (-)										5
mycophenolic acid (-)										5
microtubule polymerization (+)										5
methyl methanesulfonate (-)										5
Low Density Lipoprotein (-)										5
Laminar shear stress (-)										5
IL6 (-)										5
IL27 (-)										5
Il1 family (-)										5
Huntington's disease (+)										5
HGF (-)										5

Supplementary table 4 - Hypotheses common in ≥ 3 experiments (cont.)

GRP (-)										5
GNRHR (-)										5
GNRH1 (-)										5
EPHB1 (-)										5
EGF (-)										5
doxorubicin (-)										5
CYP2C18 (-)										5
cyclic GMP (-)										5
CSF2 (-)										5
Collagenase family (-)										5
AFP (-)										5
Act (-)										5
ABL1 (-)										5
GLI1 (-)										5
GDF2 (-)										5
concanavalin a (-)										5
benzyloxycarbonyl-Leu-Leu-Leu aldehyde (+)										5
Wnt family Mm (-)										4
TRIB3 (+)										4
topotecan (-)										4
TNFSF11 (-)										4
THPO (-)										4
TGFBR2 (-)										4
SU6656 (+)										4
sphingosine-1-phosphate (-)										4
sodium chloride (-)										4
SNAI1 (+)										4
RU486 (+)										4
response to oxidative stress (-)										4
response to endoplasmic reticulum stress (-)										4
response to DNA damage stimulus (-)										4
RAF1 (-)										4
PTGS2 (-)										4
prostaglandin E2 (-)										4
Platelet Derived Growth Factor Complex family Hs (-)										4
phenylephrine (-)										4
NTF3 (-)										4
NRAS (-)										4
NR5A2 (-)										4
nifedipine (+)										4
Luteinizing Hormone Complex Mm (-)										4
L-glutamic acid (-)										4
leukotriene D4 (-)										4
ischemia (-)										4
INHBA (-)										4
IL1B (-)										4
hyperosmotic response (-)										4
hydrogen peroxide (-)										4
HTT (-)										4
HIF (+)										4
herbimycin (+)										4
Gh (-)										4
GCG (-)										4
FOXL2 (-)										4
forskolin (-)										4
Fgf (-)										4
ERK1/2 family (-)										4
Erk family (-)										4
electrical stimulus (-)										4

Supplementary table 4 - Hypotheses common in ≥ 3 experiments (cont.)

dimethyl sulfoxide (-)										4
DCN (-)										4
Cytokine family (-)										4
cyclic AMP (-)										4
CREB1 (-)										4
complexOf(IL12A,IL12B) (+)										4
Colforsin (-)										4
cold stimulus (-)										4
CHUK (-)										4
CEBPD (-)										4
calphostin C										4
bisphenol A (-)										4
BHLHE40 (+)										4
bexarotene (+)										4
beta-estradiol (-)										4
BDNF (-)										4
ANGPT2 (-)										4
Angiotensin ii (-)										4
Alpha catenin family (+)										4
AKT3 (-)										4
A23187 (-)										4
8-bromo-cAMP (-)										4
3,3'-diindolylmethane (-)										4
STAT1 (-)										4
Stanolone (-)										4
SB-431542 (+)										4
PROX1 (-)										4
propylthiouracil (-)										4
PRL (-)										4
PARP9 (-)										4
neuron differentiation (+)										4
HOXA13 (-)										4
GNAQ (-)										4
GNA14 (-)										4
FSHB (-)										4
cocaine (-)										4
NRG1 (-)										3
ZFP36 (+)										3
VEGFA (-)										3
tunicamycin (-)										3
TRH (-)										3
Tretinoin (-)										3
trapoxin (-)										3
TNF (-)										3
thapsigargin (-)										3
Tetradecanoylphorbol acetate (-)										3
taurocholic acid (-)										3
t(ZNF217) (+)										3
t(ZBTB17) (-)										3
sulindac sulfide (-)										3
S-nitroso-N-acetylpenicillamine (-)										3
seizures (-)										3
response to osmotic stress (-)										3
response to cold (-)										3
RASSF1 (-)										3
quisqualic acid (-)										3
PTEN (+)										3
progesterone (-)										3
PRKCB (-)										3

Supplementary table 4 - Hypotheses common in ≥ 3 experiments (cont.)

plicamycin (-)										3
PKC Family Hs (-)										3
PKA family Rn (-)										3
palmitic acid (-)										3
Oxidopamine (-)										3
oxidative stress (-)										3
OSM (-)										3
nicotine (-)										3
MYC (+)										3
monocyte adherence (-)										3
MEOX2 (-)										3
lysophosphatidic acid (-)										3
L-triiodothyronine (-)										3
LTBR (-)										3
low calorie diet (+)										3
KRAS (-)										3
KLF7 (-)										3
JAK1 (-)										3
IRF5 (-)										3
insulin (-)										3
immobilization stress (-)										3
IKK family Hs (-)										3
IKBKB (-)										3
IFNG (-)										3
hypoxia (-)										3
hyperoxia (-)										3
histamine (-)										3
high frequency electrical stimulus (-)										3
Helicobacter pylori infection (-)										3
heat shock (-)										3
GSK3B (-)										3
FOXO1 (-)										3
FGFR1 (-)										3
FGF2 (-)										3
experimentally-induced arthritis (-)										3
ERK family (-)										3
EPO (-)										3
ELAVL1 (-)										3
darkness (-)										3
cycloheximide (-)										3
CTGF (-)										3
CP-31398 (-)										3
colchicine (-)										3
cell senescence (-)										3
CEBPB (-)										3
BRMS1 (-)										3
BMPR1B (+)										3
bacterial infection (-)										3
ATF4 (-)										3
AREG (-)										3
arachidonic acid (-)										3
APOE (+)										3
AP-1 complex Hs (-)										3
aldosterone (-)										3
AGN194204 (-)										3
AGN193109 (+)										3
6-cyano-7-nitroquinoxaline-2,3-dione (+)										3
2-cyano-3,12-dioxoolean-1,9-dien-28-oic acid (-)										3
15-deoxy-delta-12,14 -PGJ 2 (-)										3

Supplementary table 4 - Hypotheses common in ≥ 3 experiments (cont.)

(4-amino-1,4-dihydro-3-(2-pyridyl)-5-thioxo-1,2,4-triazole)copper(II) (-)										3
(±)-AMPA (-)										3
urethane (+)										3
troglitazone (-)										3
TNFSF10 (-)										3
TGFA (-)										3
SREBF2 (-)										3
SREBF1 (-)										3
SRC (-)										3
SP1 (-)										3
SOCS3 (+)										3
SMAD4 (-)										3
S100B (-)										3
RARB (-)										3
RAR Family Hs (-)										3
pulmonary fibrosis (+)										3
prednisolone (-)										3
NEUROG3 (-)										3
NADPH oxidase complex Mm (+)										3
MYF5 (-)										3
IFNA2 (-)										3
heat (+)										3
GAST (-)										3
fusionOf(Ret,Ncoa4) (-)										3
FAS (+)										3
EGR2 (-)										3
E. coli B4 lipopolysaccharide (-)										3
CXCR7 (+)										3
CREBBP (-)										3
cis-urocanic acid (-)										3
CAMK4 (-)										3
BTC (-)										3
BMP7 (-)										3
BMP4 (-)										3
AHR (-)										3
ADIPOQ (-)										3
